# Uncertainty Modulates the Effect of Transcranial Stimulation Over the Right Dorsolateral Prefrontal Cortex on Decision-Making Under Threat

**DOI:** 10.3389/fnins.2019.00305

**Published:** 2019-04-02

**Authors:** Jingjing Pan, Chengkang Zhu, Jianbiao Li

**Affiliations:** ^1^China Academy of Corporate Governance, Business School, Nankai University, Tianjin, China; ^2^Reinhard Selten Laboratory, Nankai University, Tianjin, China; ^3^School of Economics, Shandong University, Jinan, China; ^4^Department of Economic and Management, Nankai University Binhai College, Tianjin, China

**Keywords:** incredible threat, credible threat, uncertain, decision-making, tDCS, rDLPFC

## Abstract

Threat is a strategy that can be used to impact decision-making processes in bargaining. Abundant evidence suggests that credible threat and incredible threat both influence the obeisance of others. However, it is not clear whether the decision-making processes under credible threat and incredible threat during bargaining involve differential neurocognitive mechanisms. Here, we employed cathodal transcranial direct current stimulation (tDCS) to deactivate the right dorsolateral prefrontal cortex (rDLPFC) to address this question while subjects allocated and reported the subjective probability of future rejection under incredible threat and credible threat. We found that application of cathodal tDCS over the rDLPFC decreased the proposer’s subjective inference of probability of rejection and the offer to the responder under incredible threat. Conversely, the same stimulation did not lead to a significant difference compared to the sham group in subjective probability and offer under credible threat. These results suggested that decision-making processes under the two types of threat during bargaining were associated with different neurocognitive substrates, because the punishment for non-compliance was uncertain under incredible threat, whereas it was certain under credible threat. We decreased activity in the rDLPFC, which is involved in decision-making processes related to bargaining under incredible threats, and observed significantly impacted behavior. The differential neurocognitive bases of subjective probability of rejection under incredible threat and credible threat resulted in different tDCS effects.

## Introduction

Bargaining is an essential part of social economic exchange. Many strategies, such as threats, are used by bargainers to impact others to derive more from the exchange. According to theories of classical economics, threat can be divided into incredible threat and credible threat. Credible threat means that the threatened person must satisfy the threatener’s desire because he/she knows that he/she will be punished if they do not comply. Conversely, incredible threat means that the threatened person does not need to satisfy the threatener’s demand because he/she does not believe they will be punished. Credible threat forces the threatened person to conform, whereas incredible threat does not (Klein and O’Flaherty, 1993; Kim, 1996). However, we feel that this view surrounding incredible threat is at odds with reality, in which the threatener sometimes claims incredible threats, and the threatened person does not always believe they will not be punished. Therefore, incredible threat is often used and demonstrated to be effective in influencing bargaining outcomes. Evidence supporting the usefulness of incredible threat derives from behavior experiments, in which several participants were more compliant under incredible threats (Straub and Murnighan, 1995; Croson et al., 2003).

Some studies explained the reason why incredible threat is effective (Witte, 1992; LeDoux, 2000; Dolan, 2002; Eimer and Holmes, 2002; Phelps, 2006). Based on the Fear Appeals theory, both incredible threat and credible threat evoke fear. Once fear is triggered in decision-making, it could alter the decision-maker’s expectations of the probability of future consequences and induce a more risk-averse choice if the consequences are uncertain (Lerner and Keltner, 2000, 2001; Loewenstein and Lerner, 2003). Since incredible threat might carry weight, the consequences of the threatened person’s actions are uncertain if he/she does not satisfy the threatener’s demand. In this condition, fear triggered by incredible threat evokes the threatened person to make more risk-averse choices and to increase his/her compliance. Unlike incredible threat, credible threat is believable because it narrows the threatener’s feasible set of actions or changes the threatened person’s payoff function to influence optimal choices (Selten, 1975). Moreover, the threatened individual perceives that punishment is certain to be administered when faced with credible threats if they do not meet the threatener’s request. Therefore, fear triggered by credible threats might not play a significant role. Thus, we proposed that the mechanisms of response to these two types of threat are different. However, little is known about whether responses to these two types of threat involve distinct psychological and neural mechanisms. Hence, this study aimed to use neuroscience techniques to explore these mechanisms.

Previous neuroscience literature focused on the neural correlates of threat-related responses. The amygdala plays an essential role in producing top-down signals on sensory pathways to influence representation of threat, and is responsible for rapid deployment of attention to threatening information (for reviews, see Bishop, 2007). Moreover, the amygdala is necessary for expression of conditioned fear (for reviews, see LeDoux, 2000). Acquisition and expression of conditioned fear can be prevented through focal amygdala infusion to decrease amygdala activity (Muller et al., 1997). A series of attentional bias and anxiety studies using functional magnetic resonance imaging (fMRI) revealed common amygdala-prefrontal circuitry underlying the processes of conditioned fear and attention to threat (Bishop et al., 2004; Bishop, 2009, for reviews, see Ochsner et al., 2012). These studies suggested that the left dorsolateral prefrontal cortex (lDLPFC) plays a regulatory role in attentional deployment since reduced activity in the lDLPFC is associated with greater activity in the amygdala. In addition, studies using high-frequency repetitive Transcranial Magnetic Stimulation (HF-rTMS) and anodal transcranial Direct Current Stimulation (tDCS) techniques over the lDLPFC, which results in increased lDLPFC activity, revealed a casual role of the lDLPFC in modification of vigilance in response to threatening information (De Raedt et al., 2010; Clarke et al., 2014; Heeren et al., 2017). In contrast, greater activation of the right DLPFC (rDLPFC) is associated with increased activation of the amygdala in response to fear (Paquette et al., 2003; Schienle et al., 2005). These findings support the concept that the rDLPFC maintains attention to threat via inhibition of attentional deployment to threat-irrelevant information or low-level threatening information (Eysenck and Derakshan, 2011; Peers et al., 2013; Sanchez et al., 2016). Two studies using bilateral-balanced tDCS over the lDLPFC and rDLPFC provided indirect evidence of the causal role of the rDLPFC in deployment of attention to threat (Ironside et al., 2016, 2017). In these studies, bilateral-balanced tDCS over the DLPFC (i.e., anodal tDCS over the lDLPFC and cathodal tDCS over the rDLPFC) significantly reduced amygdala activation and attention to threat in a dot probe detection task. However, bilateral-unbalanced tDCS over the lDLPFC did not have significant effects. These outcomes indicated that cathodal tDCS over the rDLPFC decreased amygdala activation and attention to threatening information.

In our study, we used a modified UG in which proposers made an allocation under credible threat and incredible threat to evaluate response to threat communication in bargaining. We aimed to distinguish the neural correlates associated with response to credible threat and incredible threat by stimulating the rDLPFC to modify attentional control of vigilance to threat. We predicted that incredible threat and credible threat both affect the distribution of allocation of the proposer in bargaining by changing beliefs regarding subjective probabilities of future rejection by the responder which would arise from altered offers. However, rDLPFC tDCS only modulated the effect of incredible threat on the proposer’s belief of the subjective probabilities of future rejection and allocation distribution, and played a minimal role when the proposer was faced with a credible threat.

## Materials and Methods

### Participants

Ninety-two healthy volunteers (48 females; between 18 and 28 years old) were recruited from all grades and campuses of Nankai University. All participants were right-handed with normal or corrected normal vision. The exclusion criteria were a history of seizures or a history of neurological or psychiatric disorders. Every participant gave written informed consent before proceeding in the experiment. All experimental procedures were approved by the Ethics Committee of the Business School of Nankai University. The study was carried out in accordance with the approved guidelines and the Declaration of Helsinki. Experimenters and participants were blind to the stimulation conditions. Participants were randomized into one of three stimulation groups: anodal (*n* = 31, 16 females), cathodal (*n* = 31, 16 females ), or sham (*n* = 30, 16 females) stimulation of the rDLPFC. Two subjects, including a man in the cathodal stimulation group and a woman in the anodal stimulation group, reported discomfort during stimulation and were excluded from further analysis. Overall, information from 90 subjects was retained for data analysis (Table 1).

**Table 1:** Demographic characteristics of the three groups.

Items	Cathodal tDCS (*n* = 30)	Sham tDCS (*n* = 30)	Anodal tDCS (*n* = 30)	*F*/χ^2^	*p*
Gender (male/female)	14/16	14/16	15/15	0.089(χ^2^)	0.958
Age	22.800(0.497)	22.767(0.361)	22.767(0.380)	0.002(*F*)	0.998
Education (under-/post-)	14/16	14/16	12/18	0.360(χ^2^)	0.835
Career experience	0.400(0.149)	0.167(0.097)	0.167(0.084)	1.413(*F*)	0.249
Major(eco-/oth-)	18/12	17/13	19/11	0.278(χ^2^)	0.870
GPA(L/M/H)	3/14/13	4/14/12	6/7/16	3.452(χ^2^)	0.485
Household income (L/M/H)	22/7/1	22/7/1	17/9/4	4.167(χ^2^)	0.384


*Chi-square test was performed on Gender, Education, Major, GPA and Household income. Education: under- = undergraduate, post- = postgraduate; Career experience: working years; Major: eco- = economic, oth- = others; GPA: L = lower 50%, M = 20–50%, H = the top 20%; Household income: L = less than 5000 CNY per month, M = 5000–10000 CNY per month, H = more than 10000 CNY per month*.

All experimental procedures were conducted in the computerized group room of the Reinhard Selten Laboratory of Nankai University (Sellab). To conduct anonymous and fully randomized experiments, the group room was segmented into several cubicles with identical computer workstations, which were interconnected and shielded from each other.

### Task

**FIGURE 1:**
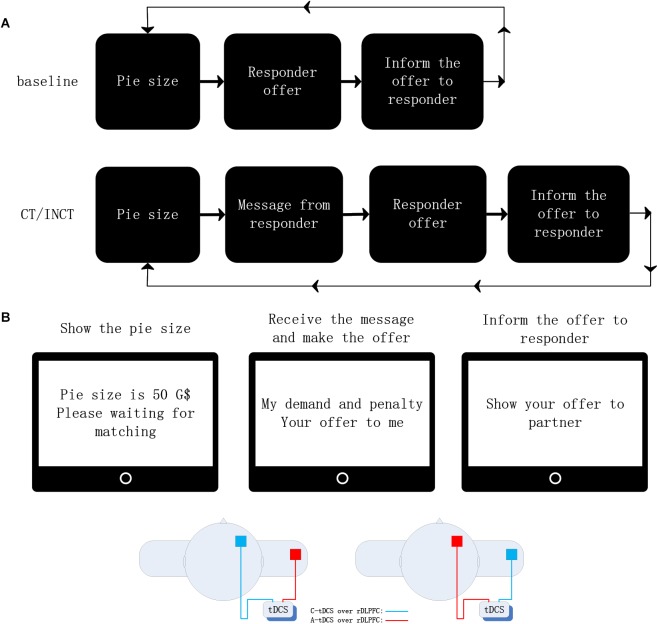
**(A)** Structure of the modified UG. UG rules: The participant made an offer to the responder after he/she received a message from the responder. They were told if the responder accepted, and the money was divided as the proposer decided. If the responder rejected, they both received zero. The message had two types of information representing two types of threat, incredible threat and credible threat (INCT and CT). With each type of threat, the requested amount included a fair request and an unfair request (fair threat and unfair threat). “Fair threat” was a 25 G$ request amount, “unfair threat” was a 35 G$ request amount. The penalty represented a deduction if the responder accepted an offer lower than his/her request amount. **(B)** The tDCS placement is shown, representing the different stimuli conditions: cathodal stimuli, cathodal electrode over the F4 site, and extra-encephalic reference on the right shoulder; anodal stimuli, anodal electrode over the F4 site, and extra-encephalic reference on the right shoulder.

The experiment was a revised ultimatum bargaining game (Figure 1), and the main unit of analysis was defined as a “round.” In the experiment, the proposer would receive a threat message from his/her matched responder in each round, making claims about the responder’s future action. Then, he or she made an offer to the responder on how to divide 50 G$ (game dollar, 1 G$ = 1 yuan); the responder could either accept (i.e., the money was divided as suggested) or reject (i.e., both proposer and responder get no money) the offer. Each round did not offer feedback about acceptance or rejection. All participants were required to play as proposers in this modified UG, and they were told that their responder would re-match after completion of a round. The participants had no information about their matched responders, and each pair interacted only through computers.

The experiment consisted of two parts. In the main paired portion (the second portion), each participant responded to 8 UG threats, including 4 incredible threats (INCT) and 4 credible threats (CT), with counter-balanced sequences. They were told that while their responders had to pay a penalty (the same as their request amount) for accepting an offer lower than their requested amount in CT, there was no penalty in the INCT condition. Therefore, they knew that if their offer did not meet the demands of the responder, they might not be rejected in the INCT condition, whereas in the CT condition they must be rejected. For both INCT and CT, the demand amount varied between 25 G$ (the fair threat) (2 rounds), and 35 G$ (the unfair threat) (2 rounds). To control for individual differences, a standard UG without communication was conducted in the first part of the experiment as a baseline treatment (2 rounds). One example of the rounds for each game was as follows: CT: “If you offer me less than 25 G$ (35 G$), I will reject your offer, otherwise I will deduct 25 G$ (35 G$)”; INCT: “If you offer me less than 25 G$ (35 G$), I will reject your offer, otherwise I will deduct 0 G$”; Baseline: “No communication in this round.”

For each experimental round, participants were presented with the threat, and they were required to make an offer within 30 s. At the same time, participants were asked to indicate the subjective probability of rejection if the offer was 1 G$ less than the demanded amount (on an 11-point Likert-scale anchored at –5 to 5, –5: surely accept, 5: surely reject) and the subjective probability of rejection if the offer was 15 G$ (on an 11-point Likert-scale anchored at –5 to 5, –5: surely accept, 5: surely reject). The first question screened the credibility of threat. The second question measured and recorded the subjective probability of rejection. Fifteen G$ was chosen on the basis of Joseph Henrich’s research on UG (Henrich, 2000). Participant indication in the first part was only in response to the second question, since the proposer could not communicate with the responder. We randomly selected one round to pay them in each part. The average payoff was 60 yuan ($9.46) (range: $6.31–$12.62, standard deviation: $1.05). The experiment was programmed in z-Tree (Fischbacher, 2007).

### Procedure and Stimuli

We recruited participants from the official accounts (Academy.org) of WeChat, BBS of Nankai University, or via e-mail. After participants were screened, we provided detailed information regarding the nature of the study, particularly the tDCS methodology. However, none of the participants were aware of the type of stimulation they received. On the day of the experiment, participants were led to an individual computer workstation. They read instructions and answered practice questions to determine that they appropriately comprehended the game. Details about how the game was played could be repeated as necessary.

A constant current flow of 1 mA was generated by a battery-driven stimulator (DC-Stimulator, NeuroConn, Germany) through a pair of a saline-soaked sponge electrode (5 cm × 7 cm; current density: 0.057 mA/cm^2^). This weak current modulates regional neural excitability by increasing or decreasing resting membrane potentials (Bindman et al., 1962). Based on the findings of Ironside et al. (2016), the “active” cathodal electrode or anodal electrode was placed over the rDLPFC, on area F4 of the international 10–20 nomenclature for EEG (Electroencephalography electrode positioning). The “reference” electrode was fixed extra-cephalically on the left shoulder (Figure 1B). The extra-cephalic reference was chosen according to Claudia Civai’s study (Civai et al., 2014) to prevent interference effects from brain areas beneath the reference electrode. Elastic bands fixed the electrodes on the head and arm, and the electrical current impedance was reduced by soaking the sponge with saline repeatedly.

In the cathodal or anodal tDCS conditions, the current was constant for 20 min with a 15 s rise and fall time, and the task started after current had been applied for 5 min. In the sham tDCS condition, the participant was only stimulated during the first and last 30 s (15 s fade-in phase, 15 s fade-out phase). If the participant reported discomfort due to stimulation, we stopped the experiment and provided compensation of 20 yuan ($7.88) as gratitude for participation. Participants practiced for 3 rounds before participating in the formal task. At the end of the experiment, participants completed questionnaires about basic demographic information (e.g., age, gender, monthly income) and a risk preference test (Holt and Laury, 2002) showing the effect of stimulation on risk attitude. This method of risk preference estimation allowed comparison of risk attitudes across a wide array of contexts and environments (Charness et al., 2013).

Moreover, it was important to specify that tDCS was not focal and that the effects of the stimulation were diffuse and not clearly confined to the area identified. However, the area under the electrode was assumed to be the area most affected by the stimulation.

## Results

To eliminate the interference of personal heterogeneity, we used the first order difference method to calculate the offer change and subjective probability change after a threat. The difference between the offer in INCT (CT) condition and that in baseline condition was defined as the offer change after incredible (credible) threat. Similarly, the difference between the subjective probability of rejection in INCT (CT) condition and that in baseline condition was defined as the subjective probability change of rejection after incredible (credible) threat.

To clarify the effect of a threat, participants’ offers and subjective probabilities of rejection in the three conditions (baseline vs. CT vs. INCT) were analyzed using paired *t*-tests. To further assess how and to what extent, tDCS influenced the effect of a threat, four mixed-design ANOVAs were used to analyze the offer change and the subjective probability change of rejection after incredible threat and those after credible threat. The between-subject factors were stimulation (cathodal, anodal, and sham), and the within-subject factors were fairness (fair threat vs. unfair threat).

Risk preference was analyzed by one-way ANOVA with stimulation (cathodal, anodal, and sham) as factor. To verify whether the participants actually distinguished between the incredible threat and credible threat in our task, we compared the credibility of incredible threat and credible threat using paired *t*-test. All tests were two-tailed, and the significance level was set at *p* < 0.05. All statistical analyses were performed in SPSS 22.0 (IBM Corp., Armonk, NY, United States).

### The Influence of tDCS on the Effect of Incredible Threat

**FIGURE 2:**
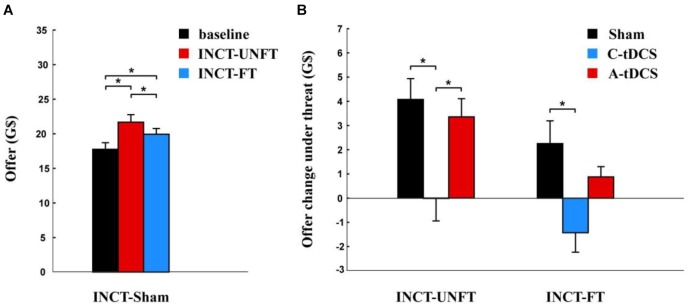
Offer change after incredible threat. **(A)** Mean offer amount to the partner and the standard error in sham group. **(B)** Effects of tDCS and fairness on the offer change and the standard error in cathodal (C-tDCS), anodal (A-tDCS), and sham stimulation group for each fairness level. The interaction between fairness and stimulation was not significant. ^∗^*p* < 0.05.

We analyzed participants’ offers in the sham group. We found that participants’ offers were significantly higher under the unfair incredible threat [mean ± SE, 21.700 ± 1.089 G$] than in the baseline condition [mean ± SE, 17.775 ± 0.954 G$, *p* < 0.001] and under the fair incredible threat [mean ± SE, 19.950 ± 0.830 G$, *p* < 0.001], and they were also higher under the fair incredible threat than in the baseline condition [*p* = 0.019] (Figure 2A).

A mixed-design ANOVA was used to analyze the influence of tDCS on the offer change after incredible threat. A significant main effect of stimulation was observed [*F* (2,177) = 8.110, *p* < 0.001, Partial η^2^ = 0.084], with a lower effect on offer change after the incredible threat in the cathodal stimulation group (mean ± SE, –0.696 ± 0.592 G$) than in the sham (mean ± SE, 3.050 ± 0.616 G$, *p* < 0.001) and anodal stimulation groups (mean ± SE, 4.675 ± 0.638 G$, *p* = 0.015). The effect on offer change after the incredible threat in the anodal stimulation group was comparable to that in the sham group (*p* > 0.1) (Figure 2B). Another significant main effect was fairness [*F* (1,177) = 18.593, *p* < 0.001, Partial η^2^ = 0.095]. The offer change was smaller after the fair threat (mean ± SE, 0.550 ± 0.431 G$) than the unfair threat (mean ± SE, 2.381 ± 0.487 G$). However, no significant interaction between fairness and stimulation was found [*F* (1,177) = 0.495, *p* > 0.1, Partial η^2^ = 0.006].

**FIGURE 3:**
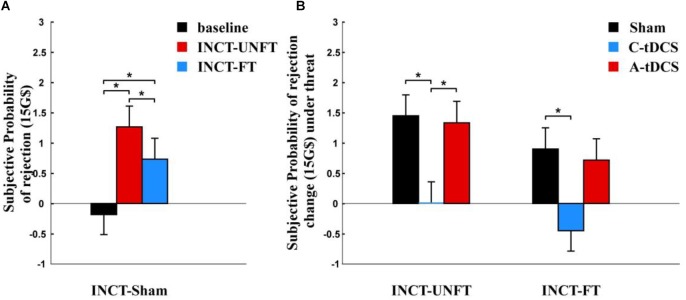
Subjective probability change of rejection after incredible threat. **(A)** Mean subjective probability of rejection and the standard error in the sham group. **(B)** Effects of tDCS and fairness on subjective probability change of rejection and the standard error in the cathodal (C-tDCS), anodal (A-tDCS), and sham stimulation group at each fairness level. The interaction was not significant between fairness and stimulation. ^∗^*p* < 0.05.

We compared the subjective probability of rejection under incredible threat with that in the baseline condition using paired *t*-tests. We found that subjective probability of rejection was significantly higher under unfair incredible threat [mean ± SE, 1.267 ± 0.344 G$] than in the baseline condition [mean ± SE, –0.1833 ± 0.331 G$, *p* < 0.001] and under the fair incredible threat [mean ± SE, 0.7333 ± 0.345 G$, *p* = 0.011]. In addition, the subjective probability of rejection was higher under the fair incredible threat than in the baseline condition [*p* = 0.011] (Figure 3A).

A mixed-design ANOVA was used to analyze the influence of tDCS on the subjective probability change of rejection after the incredible threat. We found a significant main effect for stimulation [*F* (2,177) = 5.475, *p* = 0.005, Partial η^2^ = 0.058] with a lower effect in the cathodal stimulation group (mean ± SE, –0.217 ± 0.240 G$) than in the sham group (mean ± SE, 1.175 ± 0.246 G$, *p* = 0.009) and the anodal stimulation group (mean ± SE, 1.025 ± 0.251 G$, *p* = 0.024). The subjective probability change of rejection in the anodal stimulation group was comparable to that in the sham group (*p* > 0.1) (Figure 3B). Another significant main effect for fairness [*F* (1,177) = 16.172, *p* < 0.001, Partial η^2^ = 0.084] was also observed. The subjective probability change of rejection after the fair incredible threat was smaller (mean ± SE, 0.389 ± 0.200 G$) than that (mean ± SE, 0.933 ± 0.200 G$) after the unfair incredible threat. No significant interaction between fairness and stimulation was found [*F* (2,177) = 0.103, *p* > 0.1, Partial η^2^ = 0.001].

### The Influence of tDCS on the Effect of Credible Threat

**FIGURE 4:**
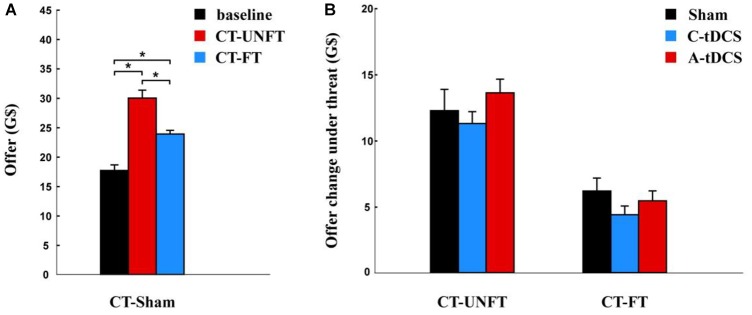
Offer change after credible threat. **(A)** Mean offer amount to the partner and the standard error in sham group. **(B)** Effects of tDCS and fairness on the offer change and the standard error in cathodal (C-tDCS), anodal (A-tDCS), and sham stimulation group for each fairness level. The interaction between fairness and stimulation was not significant. ^∗^*p* < 0.05.

We analyzed participants’ offers in the sham group. Participants’ offers were significantly higher under the unfair credible threat [mean ± SE, 30.150 ± 1.357 G$] than in the baseline condition [mean ± SE, 17.775 ± 0.954 G$, *p* < 0.001] and under the fair credible threat [mean ± SE, 24.0083 ± 0.628 G$, *p* < 0.001], and they were also higher under the fair incredible threat than in the baseline condition [*p* < 0.001] (Figure 4A).

A mixed-design ANOVA was used to analyze the influence of tDCS on the offer change after credible threat. There was only a significant main effect for fairness [*F* (1,177) = 206.091, *p* < 0.001, Partial η^2^ = 0.583]. The average offer change was smaller after the fair threat (mean ± SE, 5.380 ± 0.471 G$) than after the unfair threat (mean ± SE 12.500 ± 0.711 G$). However, no significant main effect of stimulation [*F* (2,177) = 0.639, *p* > 0.1, Partial η^2^ = 0.007] (Figure 4B) or interaction between fairness and stimulation were found [*F* (2,177) = 0.495, *p* > 0.1, Partial η^2^ = 0.006].

**FIGURE 5:**
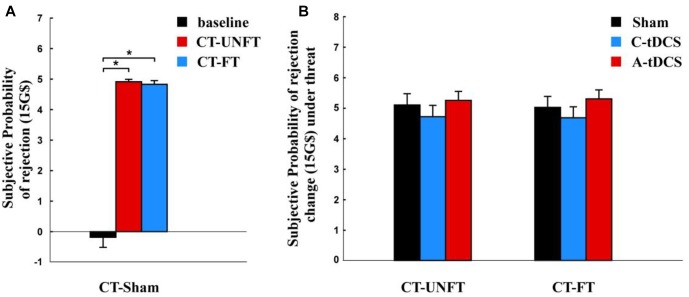
Subjective probability change of rejection after credible threat. **(A)** Mean subjective probability of rejection and the standard error in the sham group. **(B)** Effects of tDCS and fairness on subjective probability change of rejection and the standard error in the cathodal (C-tDCS), anodal (A-tDCS), and sham stimulation group at each fairness level. The interaction was not significant between fairness and stimulation. ^∗^*p* < 0.05.

We compared the subjective probability of rejection under credible threat with the baseline condition using paired *t*-test. The subjective probability of rejection was significantly higher under the unfair credible threat [mean ± SE, 4.917 ± 0.043 G$] and under the fair credible threat [mean ± SE, 4.833 ± 0.093 G$] than in the baseline condition [mean ± SE, –0.1833 ± 0.331 G$, *p* < 0.001] (Figure 5A).

A mixed-design ANOVA was used to analyze the influence of tDCS on the subjective rejection change of rejection after the credible threat. There was no significant main effect for stimulation [*F* (2,177) = 0.807, *p* > 0.1, Partial η^2^ = 0.009] (Figure 5B). Moreover, we did not find any significant main effect for fairness [*F* (1,177) = 0.319, *p* > 0.1, Partial η^2^ = 0.002] or interaction between fairness and stimulation [*F* (2,177) = 0.976, *p* > 0.1, Partial η^2^ = 0.011].

### tDCS Effects on Risk Preference

Data from the risk preference test were analyzed by one-way ANOVA. We did not find a significant main effect of stimulation [*F* (2,177) = 1.696, *p* > 0.1]. Neither cathodal [mean ± SE, 11.557 ± 0.641 G$, *p* > 0.1] nor anodal [mean ± SE, 12.867 ± 0.579 G$, *p* > 0.1] stimulation impacted risk attitude compared to sham [mean ± SE, 11.467 ± 0.577 G$]. These results suggested that the proposer’s behavioral data were not influenced by personal risk preference.

### Credibility of Threat

We compared the credibility of incredible threat and credible threat using paired *t*-tests. The credibility of credible threat [mean ± SE, 4.806 ± 0.034 G$] was significantly higher than that of incredible threat [mean ± SE, –3.178 ± 0.175 G$, *p* < 0.001].

## Discussion

The main aim of this study was to distinguish the neural correlates of responses to credible threat and incredible threat. We performed an experiment to explore the involvement of the rDLPFC under incredible and credible threat using tDCS, a technique that allowed modulation of cortical activation. We confirmed our hypothesis that cathodal tDCS of the rDLPFC decreased both subjective probability of rejections and offers under incredible threat but not under credible threat. Moreover, our results suggested that the differential neurocognitive basis of reforming the subjective probability under incredible threat and credible threat enabled the tDCS effects.

Both incredible threat and credible threat significantly increased the subjective probabilities and offer amounts compared with the baseline condition. These results demonstrated that appearance of messages which included incredible threat or credible threat information impacted allocations by increasing subjective probability of rejection.

Unfair incredible threats are significantly different from fair incredible threats, resulting in greater offer amounts. The subjective probability of rejection was also higher in response to unfair incredible threats than in response to fair incredible threats. These findings, consistent with Rankin (2003), indicated that as more value is required by the threat, the subjective probability of rejection increased. Hence, the threatened person offered more under unfair incredible threat than under fair incredible threat. Unfair credible threats resulted in greater offer amounts as well. However, the subjective probability of rejection was comparable in response to unfair and fair credible threats. This was because the subjective probability of rejection under fair credible threats had already been close to 100%.

Under incredible threat, the subjective probability of rejection varied (unfair incredible threat: 18.89% chose 4 or 5 in subjective probability of rejection; fair incredible threat: 11.68% chose 4 or 5 in subjective probability of rejection). Under credible threat, the subjective probability of rejection was identical among most individuals (unfair credible threat: 98.89% chose 4 or 5 in subjective probability of rejection; fair credible threat: 97.22% chose 4 or 5 in subjective probability of rejection). This finding indicated that the threatened person was uncertain of future rejection under incredible threat but was certain of future rejection under credible threat, if their offer amount was less than the threatener’s request. This finding was in accordance with a very early common finding that individuals’ subjective probability may deviate from objective probability unless the outcome is certain (Monat et al., 1972). Consistently, our data in response to credibility of threat also showed that individuals were able to distinguish incredible threat and credible threat in our task.

The effects of tDCS mainly showed that cathodal stimulation over the rDLPFC under incredible threat led to a prominent difference compared with the sham condition in distribution and subjective probability of rejection. Offer amount and subjective probability were dramatically lower in the cathodal condition. Interestingly, cathodal stimulation under credible threat did not result in a significant discrepancy in the subjective probability of rejection and its distribution compared to the sham group. This stimulation did not significantly impact individuals’ subjective probability of rejection under credible threat. Hence, the rDLPFC is only recruited under processing of subjective probability under incredible threats. The neurocognitive mechanisms of subjective probability under credible threat did not involve the rDLPFC.

In previous fMRI and tDCS studies (Paquette et al., 2003; Schienle et al., 2005; Bishop, 2009; Eysenck and Derakshan, 2011; Ironside et al., 2016, 2017), decreased activation of the rDLPFC specifically reduced attentional control of threat-related fearful material and prevented acquisition of threat-related fear. When we reduced participants’ vigilance to threat and made them insensitive to threat-related fear through cathodal stimulation applied over the rDLPFC (cathodal stimulation group), the threatened person’s subjective probability of rejection decreased significantly only under incredible threat but not under credible threat. These results indicated that threat-related fear might only participate in the process of generating subjective probability of rejection under incredible threat.

Under incredible threat, the subjective probability of rejection would be affected by immediate emotion such as fear in decision-making (Loewenstein and Lerner, 2003; Lin et al., 2016; Chiu et al., 2018). Fear of rejection caused by the threatener’s incredible threat likely prevented the subject from thinking rationally (Schotter et al., 1994; Van Dijk and Vermunt, 2000; Fellner and Güth, 2003) since the probability of future rejection was uncertain. This specific immediate emotion triggers under- or over-scrutiny of information (e.g., Bless et al., 1996), and individuals are more likely to make relatively pessimistic risk judgments followed by risk-averse choices (Lerner and Keltner, 2000, 2001). Since the threatened person overestimates the probabilities of disadvantageous outcomes, he/she makes a greater offer under incredible threat (e.g., Schotter et al., 1994; Straub and Murnighan, 1995; Croson et al., 2003; Carpenter and Matthews, 2004). Under credible threat, being certain of rejection when the offer is lower than the threatener’s request caused the threatened person’s subjective probability of rejection to increase to 100%, resulting in a higher offer amount compared to baseline. Credible threats should be believed (e.g., Smoke, 1987), and the threatened person should adjust their actions according to the credible threat. Therefore, fear is unable to influence the inference of subjective probability and allocation under credible threat.

Under incredible threat, the probability of rejection is uncertain, and fear may lead to overestimation of threat and likelihood of rejection (Bless et al., 1996; Lerner and Keltner, 2000, 2001). However, cathodal stimulation prevented the effect of threat-related fear on the subjective probability of the threatened person, and his/her subjective probability of future rejection by the threatener did not increase as observed in the sham group. These results suggested that individuals in this group did not experience fear-induced changes in response to threat. When incredible threat triggered fear, the subjective probability of rejection increased, and influenced them to make a larger offer. If we successfully reduced attentional control for threat-related fearful material using stimulation, the influence of fear on subjective probability would be reduced. Therefore, the subjective probability and allocation under incredible threat in the cathodal stimulation group were significantly different than those in the sham group. Under credible threat, the lack of difference in the subjective probability of rejection compared to that in the sham condition may have resulted from the proposer updating the subjective probability of rejection directly in response to threat information to reflect a belief that they would be rejected in 100% of cases if the offer was lower than the demands of the threatener. Regardless of whether the threatened person felt fear, credible threat influenced decision-making by instilling a certain subjective probability of rejection if the offer did not comply with the threat. Fear no longer factored into subjective probability, and attentional control of fear by the rDLPFC no longer had an effect.

Threat may also evoke anger and disgust, but not fear. Disgust suppresses sensory perceptual and attentional processing of disgust information to minimize contact (McNally, 2002; Krusemark and Li, 2011). This outcome suggests that if the dominant emotion in our experiment was disgust, individuals would pay little attention to the threat information and divide the pie at baseline levels. Fear and anger also have different effects. People express pessimistic risk estimates and make risk-averse choices under fear conditions, whereas they express optimistic risk estimates and risk-seeking choices under anger conditions (Lerner and Keltner, 2001). Anger evokes a fight, and fear leads individuals to want the fear-inducing stimulus to go away (Skitka et al., 2006). If anger was the dominant emotion in our experiment, individuals would have offered less than the baseline levels.

To control for other aspects of choice behavior that may be affected by stimulation, we measured the preference of risk,as described by Holt and Laury (2002), between subjects because the DLPFC is associated closely with response to risk (Bechara et al., 1996; Kuhnen and Knutson, 2005;Rao et al., 2008). In light of our results, cathodal or anodal stimulation over the rDLPFC did not impact risk attitude (Fecteau et al., 2007a,b; Yaple et al., 2017). In addition, previous studies showed that the DLPFC correlates with belief of inference or mental states in relation to others (Goel and Dolan, 2004; Costa et al., 2008; Kato et al., 2009; Yoshida et al., 2010). We controlled for the stimulation effect on belief alteration, and our results for subjective probability of rejection amounts in CT were not significantly different between subjects. This indicated that the subjective probability and choice behavior of participants was not directly affected by stimulus over the rDLPFC, which agreed with Yoshida et al. (2010). Moreover, since previous experimental studies showed that fairness of threat influenced the threatened person’s actions, we manipulated the fairness of credible threat and incredible threat by evaluating fair and unfair threats in both credible and incredible threat conditions (Rankin, 2003). Our results demonstrated that fairness of threats did not impact the effect of tDCS. Attentional control of threat in the rDLPFC refers to attentional control of threat-related negative affection or threat-related processing (Ochsner et al., 2004; Kaplan et al., 2007; Amodio et al., 2008) and not the message of threat being ignored. Therefore, the subjective probability of rejection and the offer in CT were not significantly different between subjects.

In response to anodal stimulation over the rDLPFC, we did not observe significant differences in between-subject factors compared with the sham group. We also showed that significant behavioral change resulted from unilateral neuromodulation of the rDLPFC. This unilateral salience might reflect a ceiling effect of attention on threat, and is consistent with the attentional control task in which vigilance to threat was reduced (Ironside et al., 2016).

## Conclusion

Few previous studies have investigated the neural correlates of credible threat and incredible threat in bargaining. Both are thought to contribute to obeisance during bargaining, but the core neural basis of changing individual subjective probability under incredible threat was different than that under credible threat. We showed that cathodal tDCS stimulation commonly used to control amygdala response to threat-related processes did not affect the subjective probability of future rejections and offers in credible threat bargaining. However, tDCS decreased subjective probability of future rejections and offers in incredible threat bargaining. These findings suggested that the effects of tDCS may result from different neurocognitive mechanisms under the two types of threats.

## Data Availability

The datasets generated for this study are available on request to the corresponding author.

## Author Contributions

All authors designed the experiments. CZ and JP carried out the experiments, analyzed the data, and wrote the manuscript.

## Conflict of Interest Statement

The authors declare that the research was conducted in the absence of any commercial or financial relationships that could be construed as a potential conflict of interest.
